# Competence of general practitioners in requesting and interpreting ECGs - a case vignette study

**DOI:** 10.1007/s12471-018-1124-2

**Published:** 2018-06-07

**Authors:** S. A. M. Compiet, R. T. A. Willemsen, K. T. S. Konings, H. E. J. H. Stoffers

**Affiliations:** Department of Family Medicine, Maastricht University, Care and Public Health Research Institute (CAPHRI), Maastricht, The Netherlands

**Keywords:** Electrocardiography, General practice, Quality of health care, Clinical competence, Diagnosis, Healthcare survey

## Abstract

**Background:**

Performing electrocardiography is common in general practice, but the quality of indication setting and diagnostic accuracy have been disputed.

**Objectives:**

To assess the competence of general practitioners (GPs) in their decision-making process with regard to recording and interpreting an electrocardiogram (ECG) and evaluating the relevance of the result for management.

**Methods:**

An online case vignette survey was performed among GPs and cardiologists (in 2015). Nine cases describing situations for which Dutch clinical guidelines recommend or advise against recording an ECG were presented. In each case, the participant had to make choices on recording an ECG, interpreting it, and using the result in a management decision. The reference standard for each ECG diagnosis was set by the expert author team.

**Results:**

Fifty GPs who interpret ECGs themselves, eight GPs who do not and 12 cardiologists completed the survey. Adherence to guidelines recommending an ECG was high for suspected atrial fibrillation, suspected arrhythmia present during consultation, including bradycardia, but much lower for progressive heart failure and stable angina. Diagnostic accuracy of GPs was best in atrial fibrillation (96%), sick sinus syndrome (85%) and old myocardial infarction (82%), but poor in left anterior fascicular block (16%) and incomplete right bundle branch block (10%). GPs often acknowledged the low relevance of the results of a non-indicated ECG.

**Conclusion:**

GPs do not fully adhere to Dutch cardiovascular guidelines on indications for recording ECGs. Diagnostic accuracy was high for atrial fibrillation, sick sinus syndrome and old myocardial infarction and poor for left anterior fascicular block and incomplete right bundle branch block.

**Electronic supplementary material:**

The online version of this article (10.1007/s12471-018-1124-2) contains supplementary material, which is available to authorized users.

## What’s new?

- Adherence of general practitioners (GPs) to guidelines recommending an electrocardiogram is high for suspected atrial fibrillation, suspected arrhythmia present during consultation, and bradycardia, but much lower for progressive heart failure and stable angina. GPs also adhere quite well to the recommendation not to record an electrocardiogram if arrhythmia is not present during consultation.
- Many GPs ignore the recommendation not to record an electrocardiogram in acute coronary syndrome, sports check-up and sudden death in a first-degree relative. In retrospect, GPs often acknowledged the low relevance of the results of a non-indicated electrocardiogram for their management decision.
- Diagnostic accuracy of the GPs is best for electrocardiograms showing atrial fibrillation, sick sinus syndrome or old myocardial infarction, and is weakest for left anterior fascicular block and incomplete right bundle branch block. Both of the latter electrocardiogram abnormalities also had most variability among cardiologists. Also, false abnormalities were described by GPs and—to a lesser extent—by cardiologists.

## Introduction

**Fig. 1:**
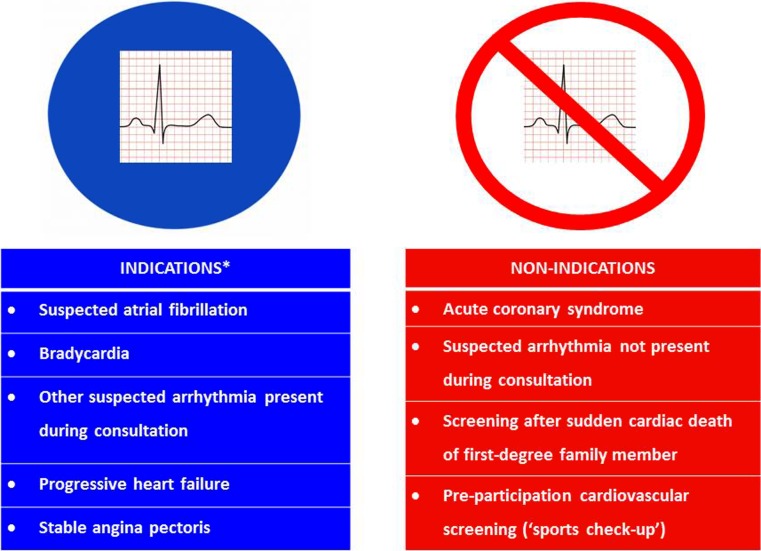
Titles of the case vignettes, grouped according to indications and non-indications for electrocardiography according to Dutch general practice guidelines [8–12]. *A case vignette on cardiovascular risk management was not included, since that topic is too complex for a vignette study [11, 18]. For a full description of all case vignettes, see Appendix (electronic supplementary material)

General practitioners (GPs) worldwide have been recording electrocardiograms (ECGs) for decades, although usefulness and diagnostic accuracy of electrocardiography by GPs have been debated frequently [1–6]. In primary care, electrocardiography may be useful as it can reduce the number of unnecessary referrals to cardiologists and it may lead to management alterations [3]. In 2014, Chan et al. summarised indications and non-indications for electrocardiography in primary care to compensate for the lack of a guideline (see Fig. 1); this raked the debate once more [4, 7]. They used current cardiovascular guidelines for GPs in the Netherlands [8–12], supplemented by international literature [7].

The quality of ECG interpretation by GPs was evaluated in several studies. These studies report that the diagnostic accuracy of GPs in interpreting ECGs is limited but varies widely. Agreement between GPs and cardiologists varied from 59–80% [3, 13, 14].

These uncertainties prompted us to design a research programme on the quality of ECGs by GPs for final year medical students (https://www.nhg.org/onderzoeken/het-ecg-de-nederlandse-huisartspraktijk-0). The aim of the current study was to assess the competence of GPs in performing (or ordering) and interpreting ECGs. To put things in perspective, we also explored how cardiologists would act in the same clinical situations.

## Methods

### Study design: a case vignette survey

We designed an online case vignette survey representing nine cases that a GP could be faced with during everyday practice [15–17]. The case vignettes described clinical situations in which there may or may not be an indication for electrocardiography, according to current literature, [8–12, 18] as summarised in the aforementioned review article (Fig. 1; [7]). Two GPs with special qualifications for cardiovascular disease, both experts in ECG interpretation (KK, RW), wrote the case vignettes [19]. The final format of the vignettes was reached after discussion and review by the research team. (For details, see electronic supplementary material).

### Recruitment of participants

Recruitment of participants took place between April and September 2015, whereas the actual survey was conducted between June 2015 and September 2015. We approached GPs who work in the three Southern Dutch provinces by email and sent reminders after two weeks. If necessary, we called the practices a week after the emails. We distributed a similar survey among acquainted cardiologists (*n* = 19) from seven cardiology departments across the Netherlands.

### Online survey

Participants had to follow a link, leading them to a web page where they could choose between ‘GP who performs and interprets ECGs’, ‘GP who does not interpret ECGs’, or ‘Cardiologist’. Then, the nine case vignettes, adapted to the participant’s category, were presented. In addition, we recorded demographic characteristics of the participants (gender, age, years of experience).

All data were exported as an Excel file, and subsequently imported in the statistics programme (IBM SPSS Statistics 21). Missing values could not occur since all fields in the questionnaire were obligatory.

### Data analysis

#### Indication setting

For each case vignette, we calculated the percentage of participants who would perform or order an ECG. Fisher’s exact test was used to compare results between groups. Free-text comments were explored for choices that deviated from the clinical guidelines [7].

#### Diagnostic accuracy

The authors’ team assessment of the ECGs was taken as reference. During the study, we became aware that the ECG with an incomplete right bundle branch block also met criteria for left anterior fascicular block, and therefore this abnormality was added as ‘correct’ answer. An incomplete right bundle branch block was defined as: an R‑R′ in V1 with a QRS width of less than 120 ms; left anterior fascicular block was defined as: a left axis, and a qR in I and/or qrS in II and III, with a QRS width of less than 120 ms [20, 21].

For each case vignette in which GPs and cardiologists made an ECG (denominator), we calculated the number of ECGs in which (a) an ECG abnormality was described in agreement with the authors’ standard (numerator); and (b) an additional abnormality was described that was not in agreement with the authors’ standard (numerator).

#### Impact of ECG results on patient management

For each case vignette, the influence of the ECG on the GP’s management decision was calculated as the percentage of ECGs marked as ‘relevant’ for the decision (numerator), compared with the total number of ECGs performed in that specific subgroup for that indication or non-indication (denominator).

## Results

### Characteristics of the participants

Before the study deadline (7 Sept. 2015), complete online questionnaires were received from 58 GPs (50 GP+ECG, and eight GP–ECG) and 12 cardiologists. The characteristics of the participating GPs are shown in the electronic supplementary Table.

### Indication setting

There were no major differences between GPs and cardiologists regarding the proportion of ECGs recorded per case vignette (Tab. 1).

**Table 1:** Number of participants in each sub-group, who would record or order an ECG for each case vignette—Fisher’s exact test comparing results of sub-groups

Title of case vignette	All participants (*N* = 70)	(a) All GPs (*N* = 58)	(b) GP-ECG (*N* = 8)	(c) GP+ECG (*N* = 50)	(d) Cardiologists (*N* = 12)	Fisher’s Exact test (c vs. d)	Fisher’s Exact test (b vs. c)	Fisher’s Exact test (a vs. d)
*Indication for ECG by GP* ^a^								
Suspected atrial fibrillation	65 (93%)	54 (93%)	*5* *(62.5%)*	*49* *(98%)*	11 (92%)	0.352	*0.007*	1.000
Bradycardia	58 (83%)	46 (79%)	5 (62.5%)	41 (82%)	12 (100%)	0.185	0.342	0.110
Suspected arrhythmia present during consultation^c^	67 (96%)	55 (95%)	*5* *(62.5%)*	*50* *(100%)*	12 (100%)	/	*0.002*	1.000
Progressive heart failure	32 (46%)	28 (48%)	1 (12.5%)	27 (54%)	4 (33%)	0.355	0.053	0.526
Stable angina	41 (59%)	32 (55%)	4 (50%)	28 (56%)	9 (75%)	0.335	0.302	0.335
*Non-indication for ECG by GP* ^**b**^								
Acute coronary syndrome	46 (66%)	37 (64%)	*1* *(12.5%)*	*36* *(72%)*	9 (75%)	1.000	*0.002*	0.526
Suspected arrhythmia not present during consultation^c^	17 (24%)	13 (23%)	2 (25%)	11 (22%)	4 (33%)	0.461	1.000	0.468
Screening after sudden death first-degree family member	46 (66%)	39 (67%)	6 (75%)	33 (66%)	7 (58%)	0.740	1.000	0.739
Pre-participation cardiovascular screening (‘sports check-up’)	42 (60%)	34 (59%)	5 (62.5%)	29 (58%)	8 (67%)	0.747	1.000	0.751

GP+ECG: General practitioners who record and interpret electrocardiograms, GP-ECG: General practitioners who do not interpret electrocardiograms (ECG)

^a^Indications: indications for an ECG according to Dutch clinical guidelines for GPs (8)

^b^Non-indications: non-indications for an ECG according to Dutch clinical guidelines for GPs (8)

^c^Other than atrial fibrillation or bradycardia

#### Indicated ECGs

All participants showed a high willingness to record an ECG for ‘suspected arrhythmia present during consultation’, ‘suspected atrial fibrillation’, and ‘bradycardia’; GPs without ECG expertise scored lowest. In the cases on ‘stable angina’ and ‘progressive heart failure’, participants would less frequently record an ECG. The free-text comments on the heart failure case showed that participants regarded clinical signals as more valuable. With regard to the angina case, GPs commented that they would not record a baseline ECG because this would be recorded directly before the exercise test anyway; they saw no need in having it done twice. In the bradycardia case, all cardiologists commented that it is important to record an ECG since the underlying condition might require further treatment.

#### Non-indicated ECGs

In the case of a ‘suspected arrhythmia not present during consultation’, indeed a minority of GPs and cardiologists would record (or order) an ECG. In the other three vignettes describing a non-indication, a majority of GPs and cardiologists would record an ECG. However, in case of an ‘acute coronary syndrome’ (ACS), the GPs without specific ECG expertise (GP–ECG) would hardly consider this. Participants who would record an ECG in this case, explained they would do this to discriminate between ST-elevation myocardial infarction (STEMI) and non-STEMI. For both ‘screening’ cases, participants commented that the ECG is often recorded as a service to the patient.

### Diagnostic accuracy

The abnormalities on the ECG that were properly diagnosed by GPs were atrial fibrillation, sick sinus syndrome, old myocardial infarction, left bundle branch block and—to a lesser extent—paroxysmal supraventricular tachycardia. Only a minority of GPs diagnosed incomplete right bundle branch block and left anterior fascicular block correctly. These abnormalities also were most difficult for cardiologists, who scored 100% for all other ECG diagnoses (Tab. 2).

**Table 2:** Number of ECGs in which an abnormality was recognised in agreement with the authors’ standard (numerator), in case vignettes in which GPs and cardiologists record an ECG (denominator)

Participant sub-group	ECG abnormality						
	Atrial fibrillation	SSS	Old infarction	iRBBB	LAFB	LBBB	PSVT (AVNRT)
*GP+ECG* *(N* *=* *50)*	47/49 (96%)	35/41 (85%)	22/27 (82%)	3/29 (10%)	3/28 (11%)	23/28 (82%)	39/50 (71%)
*Cardiologists* *(N* *=* *12)*	11/11 (100%)	12/12 (100%)	4/4 (100%)	3/8 (38%)	2/8 (25%)	9/9 (100%)	12/12 (100%)

GP+ECG: General practitioners who perform and interpret electrocardiograms

*ECG* electrocardiography, *SSS* sick sinus syndrome, *iRBBB* incomplete right bundle branch block, *LAFB* left anterior fascicular block, *LBBB* left bundle branch block, *PSVT* paroxysmal supraventricular tachycardia, *AVNRT* atrioventricular nodal re-entry tachycardia

The GPs scored false abnormalities in 12–54% of the ECG cases they interpreted (average 29%). In almost all vignettes (except paroxysmal supraventricular tachycardia), there was at least one cardiologist who described additional abnormalities that were neither described by his/her colleagues nor by the author team (average 16%) (Tab. 3).

**Table 3:** Number of electrocardiograms in which an additional abnormality not in agreement with the authors’ standard was described (numerator), in case vignettes in which GPs and cardiologists recorded an ECG (denominator)

Participant sub-group	ECG abnormality						
	Atrial fibrillation	SSS^a^	Old infarction^b^	iRBBB or LAFB	LBBB^c^	PSVT (AVNRT)	Normal ECG^d^
*GP+ECG* *(N* *=* *50)*	12/49 (25%)	22/41 (54%)	11/27 (41%)	4/29 (14%)	14/28 (50%)	15/50 (29%)	10/81 (12%)
*Cardiologists* *(N* *=* *12)*	4/11 (36%)	3/12 (25%)	1/4 (25%)	1/8 (13%)	1/9 (11%)	0/12 (0%)	2/20 (10%)

GP+ECG: General practitioners who record and interpret electrocardiograms

*ECG* electrocardiogram, *SSS* sick sinus syndrome, *iRBBB* incomplete right bundle branch block, *LAFB* left anterior fascicular block, *LBBB* left bundle branch block, *PSVT* paroxysmal supraventricular tachycardia, *AVNRT* atrioventricular nodal re-entry tachycardia

^a^In the bradycardia case, the SSS was often interpreted as a first-degree atrioventricular block, but PQ time was exactly 0.2 s

^b^The ECG in the heart failure vignette showed an old inferior and anterior/anteroseptal infarction, characterised by Q waves in leads II, III, avF and a QS complex in V3

^c^In the LBBB ECG, the false abnormalities that were most often described were conduction disorders or ST-segment alterations

^d^Three ECGs were without abnormalities; interpretation of these three ECGs is shown in the same column

### Relevance of ECG results for patient management

**Fig. 2:**
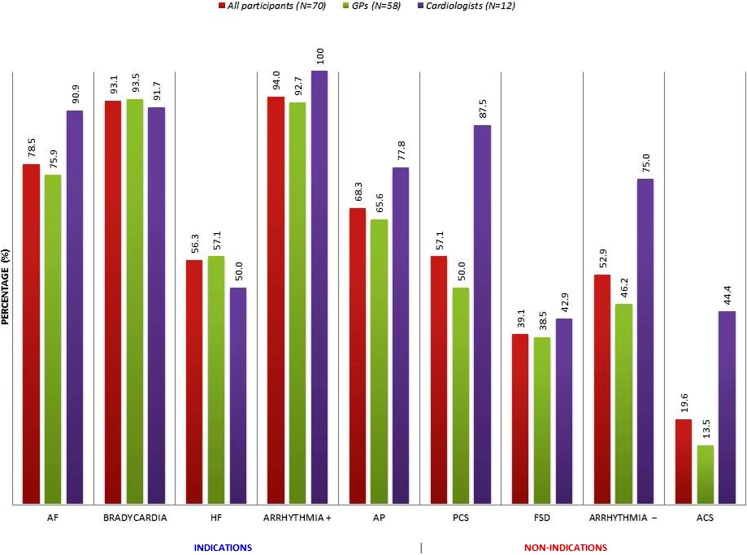
ECGs indicated as relevant for management decisions, as percentage of electrocardiograms recorded (or ordered) for each indication, according to all participants, general practioners and cardiologists, respectively. *AF* atrial fibrillation, *HF* heart failure, *AP* stable/unstable angina pectoris, *PCS* pre-participation cardiovascular screening, *FSD* familial sudden cardiac death, *ACS* acute coronary syndrome, arrhythmia + vs. −: present versus not present during the consultation

The impact of ECG results on management decisions is shown in Fig. 2. In cases with a recommendation for electrocardiography, the ECG if recorded was considered highly relevant for patient management of patients suspected of atrial fibrillation, bradycardia or another arrhythmia present during consultation. The relevance of an ECG in the heart failure and angina cases scored relatively low among both disciplines. For all case vignettes with no indication for electrocardiography, GPs considered the ECG results relevant for their management decision in a minority of the ECGs they had recorded or ordered. One GP would not refer the ACS case, motivated by a normal ECG. Cardiologists scored the impact of non-indicated ECGs relatively high, in particular, the ‘pre-participation ECG’ and ‘arrhythmia not present during the consultation’ (Fig. 2).

## Discussion

### Main findings

The participating GPs agreed with the recommendations for electrocardiography in case of possible atrial fibrillation, bradycardia and arrhythmia’s present during consultation. They also adhered quite well to the recommendation not to record an ECG in case of an arrhythmia not present during consultation. Many GPs ignored the recommendation not to record an ECG in ACS, a sports check-up and sudden death in a close relative. However, many of them admitted that the ECG results in these cases did not help them much in the subsequent decision. The diagnostic accuracy of the GPs was best in atrial fibrillation, sick sinus syndrome and old myocardial infarction, and weakest in left anterior fascicular block and incomplete right bundle branch block. Both latter ECG abnormalities had most variability among cardiologists as well. The GPs scored false abnormalities in one third of the ECGs they interpreted; the cardiologists did so in one sixth of interpreted ECGs.

### ECGs: indications and relevance

#### Progressive heart failure and stable angina

In the progressive heart failure and stable angina cases, about half of the GPs would not record an ECG, but around 60% of the ECGs that were recorded in these cases were considered relevant for a management decision. In both cases, the cardiologists would act differently: in the heart failure case, they less often made an ECG and they less often considered the results relevant, whereas in the stable angina case they moved in the opposite direction. A possible explanation for this difference is the cardiologist’s easy access to echocardiography (making an ECG less relevant) and exercise testing (needing an ECG at rest), respectively. Identifying underlying causes at the initial diagnosis of heart failure and at any moment when heart failure is worsening is essential, since this may imply therapeutic consequences. Then, an ECG is often helpful [7]. However, in case of progressive heart failure, GPs also could consider the option of open access echocardiography [22]. In the case of stable angina, GPs should realise that there are patients who may gradually develop ECG abnormalities, e.g. conduction problems, labile ST-segment, silent myocardial infarction, or left bundle branch block. These findings can be relevant for prognosis and treatment, or for indication and interpretation of the exercise ECG [7, 23]. Furthermore, not every institution performs a standard resting ECG before the exercise test.

#### Acute coronary syndrome

 GPs agreed with the point of view that in acute coronary syndrome no ECG result should keep them from deciding to refer the patient immediately. Furthermore, the ambulance nurse will record an ECG in any event. If the patient is stable, and there is time, the GP could record an ECG to prepare a possible referral to an intervention cardiologist [7]. Local working agreements between GPs, cardiologists and the ambulance organisation could preclude unnecessary ECGs. One participating GP ruled out acute coronary syndrome based on a normal ECG, which can lead to missing acute coronary syndrome [7]. Such misconceptions should be addressed in ECG education for GPs.

#### Screening ECGs

In both screening cases, GPs admitted in retrospect that the ECGs had not been very useful. If GPs want to provide ‘Service to the patient’ they rather explain to the patient that interpreting such an ECG requires specific expertise than record the ECG. Making the screening ECG and include it in the referral letter to the cardiologist or specialist in sports medicine, may be a practical compromise [24–27].

### Diagnostic accuracy

Despite the good performance of GPs in recognising atrial fibrillation, sick sinus syndrome, old myocardial infarction, left bundle branch block and paroxysmal supraventricular tachycardia (which is consistent with current literature) [2, 3, 5, 6, 14, 28], many false abnormalities were seen, and incomplete right bundle branch block and left anterior fascicular block were frequently missed. These QRS abnormalities have few clinical consequences, and the criteria are much more susceptible to inter-individual interpretation [20, 21]. Diagnostic accuracy can be maintained by continued systematic assessment of many ECGs, and by more often asking a colleague (or a cardiologist) to assess the ECG as well.

Our study revealed that also cardiologists show variability in their assessments of ECGs. This implies that in research, a panel of two or three independent ECG experts, who need to reach consensus in case of disagreement, may be necessary.

### Strengths and limitations of this study

#### Free-text comments

The free-text comments on the motives for recording of ECGs and the relevance of ECG results allowed us to make nuanced interpretations of the participants’ choices that departed from the guidelines.

#### Participants

The low number of GPs who do not interpret ECGs themselves limited the statistical comparison of the sub-groups in our study. Percentages regarding false additional ECG abnormalities should be viewed with caution since some ECGs were evaluated by a very small number of participants. It is uncertain whether our southern Dutch GP population was representative of the Netherlands. Our GP group included more women and was younger than the practicing GP population in the Netherlands; the proportion of single-handed GPs (21%) was similar to this proportion in the Netherlands (22%) [29].

#### Reference standard

There was full consensus between the author team and all cardiologists on the ECG diagnoses atrial fibrillation, sick sinus syndrome, old myocardial infarction, left bundle branch block, and paroxysmal supraventricular tachycardia, respectively. It became apparent that cardiologists do not always agree among each other (Tab. 3).

### Conclusions

Adherence of GPs to guidelines recommending ECG was high for suspected atrial fibrillation, suspected arrhythmia present during the consultation, and bradycardia, but much lower for progressive heart failure and stable angina. In retrospect, GPs often acknowledged the low relevance of the results of a non-indicated ECG for their management decision. Diagnostic accuracy of ECGs by GPs was best in atrial fibrillation, sick sinus syndrome and old myocardial infarction, and poorest in incomplete right bundle branch block and left anterior fascicular block. False abnormalities were described by GPs and—to a lesser extent—by cardiologists.

## Caption Electronic Supplementary Material

ESM1: Extra Table - Characteristics of participating GPs in a case vignette survey on ECG indications and interpretation, per subgroup

ESM2: Appendix – Case Vignettes
