# Supplementary material for: Competence of general practitioners in requesting and interpreting ECGs - a case vignette study
Source: Neth Heart J. 2018 Jun 7;26(7-8):377–84. doi: 10.1007/s12471-018-1124-2 (PMC6046661; doi:10.1007/s12471-018-1124-2)
Supplement: Supplementary file 1 — ESM1: Extra Table - Characteristics of participating GPs in a case vignette survey on ECG indications and interpretation, per subgroup [file 12471_2018_1124_MOESM1_ESM.docx]

Online Supplement

**Extra Table** Characteristics of participating GPs in a case vignette survey on ECG indications and interpretation, per subgroup

| ***GP Characteristics*** | | ***GP+ECG***  ***(N=50)*** | ***GP-ECG***  ***(N=8)*** |
| --- | --- | --- | --- |
| *Gender* | Male | 24 | 3 |
|  | Female | 26 | 5 |
| *Age group (in years)* | 26-35 | 12 | 1 |
|  | 36-45 | 11 | 3 |
|  | 46-55 | 12 | 1 |
|  | >55 | 15 | 3 |
| *Practice* | Single-handed practice | 10 | 2 |
|  | Group practice | 40 | 6 |
| *Experience as GP (in years)* | 1-5 | 8 | 1 |
|  | 6-10 | 8 | 1 |
|  | 11-15 | 11 | 2 |
|  | 16-20 | 10 | 0 |
|  | >20 | 13 | 4 |
| *Experience with ECG (in years)* | 1-5 | 17 |  |
|  | 6-10 | 14 |  |
|  | 11-15 | 9 |  |
|  | 16-20 | 3 |  |
|  | >20 | 7 |  |

*GP+ECG* General practitioners who make and interpret electrocardiograms, *GP-ECG* General practitioners who do not interpret electrocardiograms themselves
