# Supplementary material for: Competence of general practitioners in requesting and interpreting ECGs - a case vignette study
Source: Neth Heart J. 2018 Jun 7;26(7-8):377–84. doi: 10.1007/s12471-018-1124-2 (PMC6046661; doi:10.1007/s12471-018-1124-2)
Supplement: Supplementary file 2 — ESM2: Appendix – Case Vignettes [file 12471_2018_1124_MOESM2_ESM.docx]

APPENDIX - CasE vignetteS (online supplement)

Each vignette started with a typical description of relevant signs and symptoms of a patient visiting the GP’s practice. Next, the participant was asked whether she/he would record (or order) an ECG in this case (yes/no) and what the motives for this decision were (free text). If an ECG was ordered and recorded, the GPs who interpret ECGs themselves (GP+ECG) and cardiologists were asked to describe and interpret it; they could choose their probable diagnosis from a given list (including an ‘Other, i.e. ...’ option). GPs who would order an ECG and have it interpreted by someone else (GP–ECG), were given the ECG conclusion. Finally, the participants were asked what their management decision would be (tick list), and if the ECG result had influenced their management decision (yes/no; free text).

***Case 1 (atrial fibrillation)***

*A 76-year-old male patient with a history of COPD Gold II and type 2 diabetes visits the GP with complaints of light-headedness, chest pressure and exertional dyspnoea since 4 days. He takes his medication, metformin 850 milligram b.i.d. and tiotropium 1 8microgram q.d., adequately.*

*You see a patient who does not appear ill and you measure a blood pressure of 130/78 mmHg and an irregular pulse of 130 beats per minute and respiratory rate of 14 per minute. Auscultation of heart and lungs sounds normal (aside from irregular heart rate). There is no oedema in the extremities.*

Een 76-jarige patiënt met in de voorgeschiedenis COPD Gold II en niet-insuline-afhankelijke diabetes mellitus type II presenteert zich met lichtheid in het hoofd, druk op de borst en dyspnoe d'effort sinds 4 dagen. Zijn medicatie bestaat uit metformine 850 mg 2dd1 en tiotropium 18 microgr 1dd1, welke adequaat worden ingenomen. De klachten treden onafhankelijk van inspanning op. De huisarts ziet een niet zieke patiënt en vindt bij lichamelijk onderzoek een bloeddruk 130/78mmHg, een polsfrequentie van 130 irregulair aequaal, ademhalingsfrequentie van 14/minuut en hoort over hart en longen geen bijzonderheden (behoudens een irregulair ritme). Er is geen oedeem in de extremiteiten.

***Case 2 (acute coronary syndrome)***

*A 48-year-old male patient presents himself at the GP’s office with chest pressure since one hour. The pain is located at the front of the thorax (on the left and on the right) and radiates to the jaw and left arm. He is nauseous and perspiring. You measure a blood pressure of 148/88 mmHg and a regular pulse of 70 beats per minute. You consider acute myocardial infarction (AMI), but have some doubts since the patient presented himself with the exact same symptoms one year earlier. Back then, he was referred to the cardiologist, where troponins were low. An additional exercise test with ECG recording was normal and no cardiac explanation for the symptoms was found.*

Een 48-jarige patiënt komt naar de huisartsenpraktijk met een pijn op de borst, sinds een uur bestaand. Patiënt geeft aan pijn te hebben aan de voorzijde van de borstkas, links- en rechtszijdig, met uitstraling naar kaken en linker arm. Hij is misselijk en hij transpireert. U meet een bloeddruk van 148/88 met een regulaire pols van 70/minuut. U overweegt een acuut coronair syndroom. U heeft enige twijfelt omdat patiënt een jaar eerder soortgelijke klachten had en met spoed naar de cardioloog werd verwezen. Bij de cardioloog bleken de troponines bij herhaling normaal en was een fietsergometrisch onderzoek zonder afwijkingen. Er is toen geen cardiale oorzaak gevonden.

***Case 3 (bradycardia)***

*An 87-year-old female patient is brought to the GP by her son. She has a history of hypertension, for which she takes hydrochlorothiazide 12,5 milligram q.d.. and metoprolol 100 milligram retard. Normally, she is active inside her home (she lives by herself), but since two days she only sits in her chair. She is tired, but does not have any other complaints. Physical examination provides you with a blood pressure of 108/78 mmHg and a pulse of 44 beats per minute, regular. Auscultation of heart and lungs is normal and there is no oedema on the extremities.*

Een patiënte van 87 wordt door haar zoon naar de huisarts gebracht. Patiënte is bekend met hypertensie, waarvoor zij al jaren hydrochloorthiazide 12,5 mg 1d1t en metoprolol 100 mg MGA 1d1t. Ze is meestal vrij actief in huis (ze woont zelfstandig, alleen), maar nu zit ze al twee dagen de hele dag in de stoel. Ze is erg moe, andere klachten heeft ze niet. Bij lichamelijk onderzoek heeft zij een bloeddruk van 108/78 mmHg, met een polsfrequentie van 44/minuut, regulair aequaal. Over hart en longen worden geen bijzonderheden gehoord en er is geen sprake van oedeem.

***Case 4 (progressive heart failure)***

*A 74-year-old male patient has a history of heart failure with a moderate mitral insufficiency and an anterior myocardial infarction 3 years ago. He uses metoprolol 100 milligram retard q.d., enalapril 10 milligram q.d., furosemide 40 milligram q.d., simvastatin 40 milligram q.d. and carbasalate calcium 100 milligram q.d. The left ventricular ejection fraction was 40% at his last check up, 1.5 years ago.*

*He now visits the GP because of orthopnoea and exertional dyspnoea since 3 days. The patient is not aware of any recent weight gain. The blood pressure is 138/78 mmHg, the pulse 88 beats per minute, regular. Auscultation of the heart shows a known holosystolic heart murmur, II/VI with point of maximal impulse on the cardiac apex. Auscultation also reveals crackles at the basis of both lungs. There is moderate oedema on the extremities.*

*The most recent letter of the cardiologist is dated 1.5 years ago and states that, apart from an old anterior myocardial infarction, no other abnormalities show on the ECG.*

Een 74 jarige patiënt is bekend met hartfalen bij een matige mitralis insufficiëntie en een voorwand infarct 3 jaar geleden. Hij gebruikt metoprolol MGA 100 mg 1d1t, enalapril 10 mg 1d1t, furosemide 40 mg 1d1t, simvastatine 40 mg 1d1t, carbasalaat calcium cardio 100 mg 1d1t. De linker ventrikel ejectiefractie was bij de laatste controle, 1,5 jaar geleden, 40%. Hij komt nu bij de huisarts i.v.m. drie dagen bestaande orthopnoe en dyspnoe d'effort. Er is enkeloedeem. Het is patiënt niet bekend of hij is toegenomen in gewicht. De bloeddruk is 138/78, de pols is 88/minuut regulair. Er is een bekende holosystolische souffle hoorbaar, met punctum maximum ter hoogte van de apex, luidheid II/VI. Over de longen bibasaal lichte crepitaties. Er is inderdaad matig enkeloedeem. In een brief van de cardioloog staat het meest recente ECG beschreven, van 1,5 jaar geleden. Er wordt o.a. gemeld dat daar een oud, vermoedelijk voorwand, infarct zichtbaar is en dat er verder geen ECG afwijkingen te zien zijn.

***Case 5 (pre-participation cardiovascular screening)***

*A 40-year-old male patient wants to run his first marathon. However, first he wants you to make an ECG to check his heart. After all, this has never been done before.*

*Up to the age of 37, he was overweight, smoked and never practiced any sports. Three years ago, he decided to change his lifestyle; he since then quit smoking and started jogging for 10 km two times a week. Now he wants to run a marathon with two friends. Both of these friends had an ECG, which were normal. The patient knows that guarantees can never be given, but he would feel a lot safer if his ECG also proves normal.*

*He has no medical history and physical examination is normal; he has a blood pressure of 126/78 mmHg with a pulse of 68 beats per minute, regular. Auscultation of heart and lungs is normal.*

Een patiënt van 40 die voor het eerst een marathon wil lopen, wenst een ECG om te zien of zijn hart in orde is. Er is immers nog nooit een hartfilmpje gemaakt.

Tot zijn 37e had hij wat overgewicht, rookte hij behoorlijk en sportte hij nooit. Daarna heeft hij het hardlopen ontdekt en nu loopt hij 2x per week 10km, is hij op een normaal gewicht gekomen en ook is hij nu al drie jaar gestopt met roken. Hij heeft nu het plan opgevat om zijn eerste marathon te lopen met zijn twee loopmaten. Die twee loopmaten hebben recentelijk allebei een ECG laten maken en dat bleek er goed uit te zien. Patiënt wil met het oog op de marathon ook graag zeker weten dat zijn hart in orde is. Hij snapt dat garantie niet bestaat, maar hij zou zich wel een stuk veiliger voelen als het ECG in orde is.

Zijn voorgeschiedenis is verder blanco en bij lichamelijk onderzoek vindt u een bloeddruk 126/78 met een pols van 68/ regulair en over hart en longen geen bijzonderheden.

***Case 6 (sudden cardiac death of first degree family member)***

*A 30-year-old male patient visits the GP. At the age of 33, his father died of sudden cardiac death during a soccer match. The patient feels healthy, has no complaints and exercises regularly. His wife urged him to go to the doctor, because ‘with such a family history you never know...’. Physical examination is without abnormalities; you find a blood pressure of 124/82 mmHg, a pulse of 64 beats per minute, regular, and auscultations of heart and lungs are normal.*

Een 30-jarige patiënt bezoekt uw spreekuur omdat hij zijn vader verloor toen hij zelf 2 was (leeftijd vader destijds 33). Zijn vader overleed aan 'hartstilstand' tijdens een voetbalwedstrijd. Patiënt voelt zich gezond en sport, zijn vrouw dringt aan op een ECG want 'je weet maar nooit' met zo'n geschiedenis. Patiënt heeft geen klachten en bij lichamelijk onderzoek vindt u een bloeddruk 124/82 met pols 64/minuut, over hart en longen geen bijzonderheden.

***Case 7 (stable angina pectoris)***

*A 73-year-old female patient with a history of hypertension, for which she takes hydrochlorothiazide 12.5 milligrams q.d., visits the GP because of flank pain and back pain during exercise since 5 months. The pain is always present during walking of short distances and disappears quickly after seizing the exercise. The GP considers angina pectoris and considers an exercise test with ECG recording. He can order this examination at the nearest hospital. Physical examinations shows no abnormalities; blood pressure is 154/86 mmHg, pulse 88 beats per minute, regular, and auscultation of heart and lungs is normal.*

Een patiënte van 73 jaar oud met in de voorgeschiedenis hypertensie waarvoor hydrochloorthiazide 12,5mg 1d1t bezoekt het spreekuur i.v.m. sinds 5 maanden pijn in de flanken en rug bij inspanning. Deze pijn treedt steevast op bij het lopen van geringe afstanden en verdwijnt snel in rust. De huisarts denkt aan angina pectoris en overweegt een fietsergometrisch onderzoek in eigen beheer. Hij kan dit zelf direct aanvragen in het nabijgelegen ziekenhuis en zal vervolgens de uitslag krijgen om deze aan patiënte mede te delen. Bij lichamelijk onderzoek vindt de huisarts een bloeddruk 154/86, pols 88/minuut regulair en over hart en longen geen bijzonderheden.

***Case 8 – (Light-headedness)***

*A 34-year-old female patient has visited your practice regularly in the past years because of her anxiety disorder. You have successfully treated her with paroxetine and psychological help. She has been without complaints for two years. However, unfortunately, she recently developed new complaints. The old complaints were blurred vision and chest pressure. Now she experiences and anxious feeling in combination with light-headedness, shortness of breath and palpitations. She had four or five of these episodes in the last few weeks. They last 15-20 minutes each time.*

*At the moment of consultation, she has no complaints and physical examination provides you with a blood pressure of 132/82 mmHg, a pulse of 72 beats per minute, regular. Auscultation of heart and lungs is normal.*

Een patiënte van 34 jaar heeft u in het verleden vaak bezocht i.v.m. een paniekstoornis en u heeft haar daarvoor ook behandeld met paroxetine en psychologische hulp. Ze was twee jaar klachtenvrij. Ze heeft nu echter weer aanvalsgewijs klachten van een angstig gevoel. Een dergelijke aanval heeft ze de laatste weken vier of vijf keer gehad, waarbij de aanval telkens 15-20 minuten duurde. Ze vertelt dat er bij de huidige klachten lichtheid in het hoofd is, kortademigheid en het gevoel van een snelle hartslag. Dit is anders dan voorheen, want destijds gingen de angstklachten gepaard met pijn op de borst en wazig zien. Op het moment van dit bezoek is ze klachtenvrij en bij lichamelijk onderzoek vindt u een bloeddruk 132/82, een pols 72/minuut regulair en over hart en longen geen bijzonderheden.

***Case 9 = case 8 continued***

*A week later, the patient visits the practice. She is having another episode, since 15 minutes. Her blood pressure is 116/78 mmHg and her pulse 164 beats per minute, regular.*

Patiënte komt een week later met klachten, nu een kwartier bestaand. De bloeddruk is 116/78 mmHg, de polsfrequentie is 164/minuut, regulair.
